# Wavelet-Prototypical Network Based on Fusion of Time and Frequency Domain for Fault Diagnosis

**DOI:** 10.3390/s21041483

**Published:** 2021-02-20

**Authors:** Yu Wang, Lei Chen, Yang Liu, Lipeng Gao

**Affiliations:** Institute of Vibration Engineering, Zhengzhou University, No. 100 Science Street, Zhengzhou 450001, China; wy1475899830@163.com (Y.W.); zzuliuy@yeah.net (Y.L.); glp_0326@163.com (L.G.)

**Keywords:** fault diagnosis, few-shot learning, meta-learning, rotating machinery

## Abstract

Neural networks for fault diagnosis need enough samples for training, but in practical applications, there are often insufficient samples. In order to solve this problem, we propose a wavelet-prototypical network based on fusion of time and frequency domain (WPNF). The time domain and frequency domain information of the vibration signal can be sent to the model simultaneously to expand the characteristics of the data, a parallel two-channel convolutional structure is proposed to process the information of the signal. After that, a wavelet layer is designed to further extract features. Finally, a prototypical layer is applied to train this network. Experimental results show that the proposed method can accurately identify new classes that have never been used during the training phase when the number of samples in each class is very small, and it is far better than other traditional machine learning models in few-shot scenarios.

## 1. Introduction

Rotating machinery plays a very important role in industrial production, but the failure of rotating equipment can bring huge economic losses to a business, and even cause casualties. Bearings are the core components of rotating machinery, therefore, it is of great significance to diagnose the fault of the bearings in time. Since the 1960s, fault diagnosis has gradually attracted scholars’ attention in the field of science and technology [1]. Traditional fault diagnosis is a diagnosis method based on fault mechanism. Nicolò et al. have studied the cracked rotor model and explained the crack mechanism [2], and Chen et al. have studied the vibration signal feature extraction method based on the failure mechanism [3]. With the continuous in-depth study of fault mechanism by scholars, the preprocessing of vibration signals, such as noise reduction, feature extraction, and other signal processing methods have also begun to develop. In [4], a multi-point optimal minimum entropy deconvolution and convolution repair method is proposed for vibration fault detection; and Pan et al. studied the symplectic geometric mode decomposition and its application in the compound fault diagnosis of rotating machinery [5].

With the development of the Internet, the field of fault diagnosis has entered the era of big data [6]. Traditional fault diagnosis methods requires high professional skills and has low diagnosis efficiency. Therefore, fault diagnosis models based on feature extraction and intelligent classification have been proposed. Early feature extraction methods include FFT (fast Fourier transform), wavelet analysis, etc. [7,8], which reduce the dimensions of the extracted features. Commonly used methods include principle component analysis (PCA) and independent component analysis (ICA), manifold learning [9,10,11], and other algorithms. At the end of the process, the dimensionality-reduced features are sent to the classifier to classify the fault. Common classifiers include neural networks, support vector machines [12,13,14], and so on. However, feature extraction algorithms rely on expert knowledge and do not have adaptive capabilities. The features extracted manually may not be the optimal solution. Therefore, a one-dimensional convolutional neural network based on automatically extracted features has been proposed and achieved good classification results [15].

**Figure 1:**
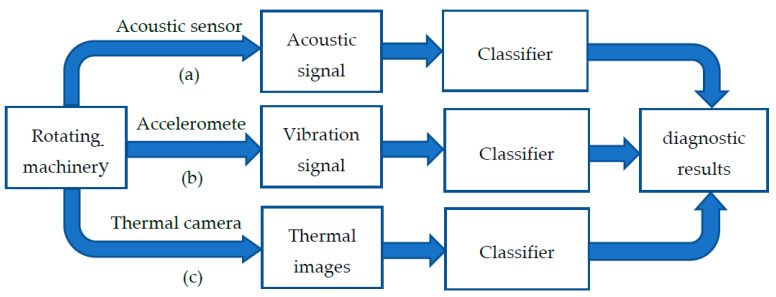
Three methods for fault diagnosis: (**a**) Acoustic analysis; (**b**) Vibration analysis (the proposed method); (**c**) Thermal images analysis.

Neural network is a data-based fault diagnosis method. According to the type of data used in its training, intelligent diagnosis methods can be divided into three types: Acoustic analysis, Vibration analysis, and Thermal images analysis (as shown in Figure 1). Acoustic analysis comprises of collecting the acoustic signal of the machine through the acoustic sensor, taking the acoustic signal as sample, and sending it to the neural network for training. A convolutional neural network that can be used for acoustic analysis for fault diagnosis of gears is proposed in [16]. Vibration analysis is the most widely used, because the vibration signal changes most obviously in the middle and late stages of the failure. The model proposed in this paper also uses vibration signals to classify faults. Neural networks are also widely used in thermal images analysis. In [17,18], the feature vectors extracted from thermal images were used to train neural networks.

The convolutional neural network has achieved high accuracy in the field of intelligent fault diagnosis, but in practical applications, once a fault occurs, the equipment will stop immediately, resulting in the inability to collect enough fault samples for the neural network [19,20]. In addition, some faults do not occur frequently, and it is difficult to collect sufficient sample data. The neural network is a data-dependent classifier [21]. Once the number of samples is insufficient, the classification accuracy will drop significantly [22]. Therefore, performing an intelligent diagnosis of rotating equipment with limited samples, is a new area to be solved [23]. There are currently two methods to solve this problem. One refers to training a model based on transfer learning, which includes pre-training and fine-tuning, or using a deep transfer network with joint distribution adaptation [24,25,26,27]. The second refers to using a few-shot learning based method. Few-shot learning is a kind of meta-learning [28]. In recent years, scholars have achieved many results in the field of meta-learning, mainly including initialization-based models, such as Model-agnostic meta-learning [29,30], and metric-based models, such as Siamese networks [23,31,32,33], matching networks [34,35], prototypical networks [36,37,38,39], etc. All of these models have good cross-domain performance and high accuracy. Among them, prototypical networks and Siamese networks are widely used in few-shot learning for intelligent diagnosis.

Wang H et al. proposed Deep Prototypical Networks, which combine the advantages of the prototypical network and the Siamese network, using the Siamese structure to extract features, and then use the prototype learning method to map this to the feature space [37]. Li B et al. use multi-scale dynamic fusion to extract features and improve the clustering method of the prototypical network for intelligent diagnosis of planetary gearbox [36]. The above studies have achieved good results, but they all use only the time domain data of the raw signal, whereas the frequency domain information is more sensitive to some faults [38]. Therefore, it is necessary to use a neural network to extract the characteristics of the frequency domain information as a supplement to the original signal. Furthermore, the prototypical layer directly maps the extracted features into the feature space without any preprocessing, which may reduce the accuracy of the neural network. To address this problem, a wavelet layer can be designed before the prototypical layer to improve the generalization of the model, which can combine the advantages of artificial neural network and wavelet analysis, that is, the network converges quickly and avoids falling into the local optimum. It also has the characteristics of time-frequency local analysis [40,41,42]. The main contributions of this paper are summarized as follows:

(1): In order to supplement the original data, a parallel two-channel convolutional structure is designed, which can receive the time domain and frequency domain information of the signal at the same time, and extract the features separately.

(2): For the purpose of further improve the generalization and accuracy of the model, a wavelet layer is proposed to preprocess the extracted features. The wavelet layer replaces the activation function of the hidden node of the neural network with a wavelet function. The bias of the hidden layer is replaced by the translation vectors and dilation vectors of the wavelet function. Combining the advantages of wavelet analysis, it can analyze the local characteristics of information flexibly. 

(3): The proposed model can accurately identify new classes that have never been used during training phases when the number of samples in each class is very small.

(4): The wavelet-prototypical network based on fusion of time and frequency domain (WPNF) is proposed, some experiments are carried out on **WPNF** and some other machine learning models. The experimental results are compared and visualized to verify the effectiveness of the proposed method. 

The remainder of this article is arranged as follows: The relevant background and terminology are introduced in section two. In section three, the structure design of the proposed model is introduced. Some experiments are carried out, and the experimental results are analyzed to evaluate our method against other methods in section four. Finally, we draw a conclusion in section five.

## 2. Related Background and Terminology

### 2.1. Meta Learning

When we learn new things, we do not need to learn from scratch, because we can use prior knowledge. As long as we have learned the method of learning, we can learn very well with few samples. Similarly, when a model starts to learn a brand new task, it does not need to start from scratch, it can learn quickly from previous experiences. In other words, let the model learn to learn by itself. This is the basic idea of meta-learning.

Suppose we have learned a series of tasks: $t_{j}\in T$, T represents the task set, which is defined by their parameters $\theta_{i}\in \Theta$, where Θ represents the parameter set. P is the set of all previous evaluation indexes, $P_{i,j}=P\left(\theta_{i},t_{j}\right)$ represents the scalar evaluations of task $t_{j}$ configured by parameters $\theta_{i}$, such as accuracy rate, cross-validation, etc. $P_{new}=\;P_{i,new}$ represents learning a new task $t_{new}$ under the known scalar evaluations $\theta_{i}$. The task of meta learning is to train a meta learner L, which can find a new parameter set $\Theta_{new}^{*}$ for the new task $P_{new}$. L should be trained on the meta-data set $P\cup^{}P_{new}$ [43]. P is usually collected in advance or extracted from the metadata repositor [44,45]. Meta learning generally learns $P_{new}$ by gradient descent method.

### 2.2. Few-Shot Learning

Few-shot learning is a type of meta-learning that aims to train a classifier to identify new classes which have never been used during the training stage, and each new class only gives a few samples. Traditional machine learning methods do not have good generalization, so using traditional neural networks for training will cause serious overfitting. The main idea to solve this problem is to let the machine learn to learn instead of just learning a specific task. The model should be like a human being. After being trained with enough tasks, when accepting a new similar task, it can achieve high performance based on previous experiences without requiring enough samples.

In order to distinguish few-shot learning from the traditional machine learning model, the data set is divided into a support set and a query set. Suppose the number of categories is C, and each category has *K* labeled samples, the support set is expressed as $S={\left\{\left(x_{i}^{s},y_{i}^{s}\right)\right\}}_{i=1}^{n_{s}}\left(n_{s}=C\times K\right)$, where $x_{i}\in {\mathbb{R}}^{D}$, and D represent the dimension of the feature vector $x_{i}$., and $y_{i}\in \left\{1,2\ldots C\right\}$ represents the label corresponding to each sample. The query set is expressed as $Q={\left\{\left(x_{i}^{q}\right)\right\}}_{i=1}^{n_{q}}\left(n_{q}=C\times L\right)$, where C represents the number of categories, and L represents the number of samples in each category.

Given a support set S, the model should correctly classify the category of each sample in the query set. The support set and the query set should contain the same classes. Suppose there are a total of C categories, and only K labeled samples in the support set of each category, this problem is called the C-way K-shot problem. *K* generally takes 1,3,5,10.

## 3. The Proposed WPNF

Traditional machine learning models have achieved excellent classification results when the sample size is sufficient, but when faced with tasks with insufficient samples, serious overfitting will occur, which leads to low generalization performance and causes the test accuracy to drop significantly. To solve this problem, we must let the model learn to learn like human beings. Prototype learning is a way of meta-learning, and its learning goal is learning how to classify, not limited to a specific classification task. That is the reason why we used a prototypical layer to train the model. We also designed a parallel two-channel convolutional structure to increase the number of input channels of the model, so that the frequency domain information of the raw signal can also be captured by the convolutional layers as a supplement to the original signal, and proposed a wavelet layer to further optimize the extracted features. Finally, the fault classification was performed by the prototypical layer.

### 3.1. The Architecture of the Proposed Model

**Figure 2:**
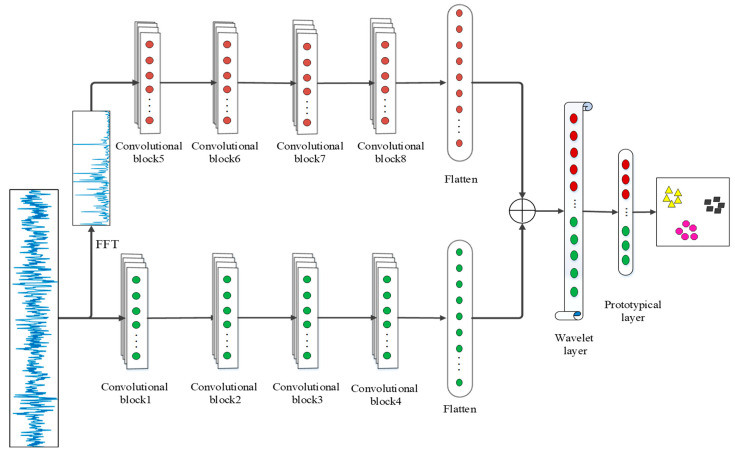
The architecture of the wavelet-prototypical network based on fusion of time and frequency domain (WPNF).

The overall structure of the model is shown in Figure 2. The raw signal is transformed by FFT transformation to obtain its frequency domain information, and then sent together to two independent channels for feature extraction (the weights of the two channels are updated separately, not shared). The features extracted by the two channels are concatenated after passing through the convolution block, and the combined feature vector is sent to the wavelet layer for further extraction. Each convolution block is composed of convolution layer, batch normalization, activation layer, and pooling layer (the first and fifth convolution blocks have a wide convolutional kernel (64 × 1) to suppress noise, the remaining convolution blocks all have a 3 × 1 convolutional kernel). The features extracted in the time domain and frequency domain will be spliced together at the ‘+’, and then sent to the wavelet layer for further processing, and finally mapped to the feature space by the prototypical layer.

### 3.2. Convolutional Block

**Figure 3:**
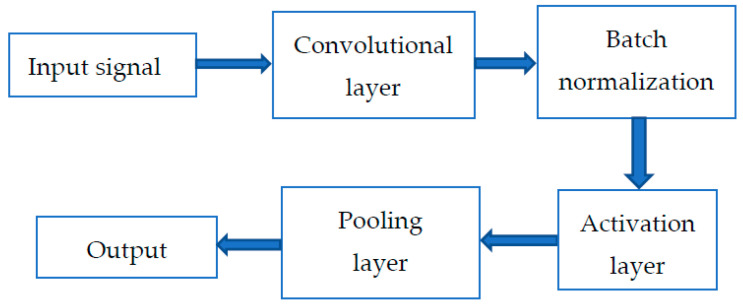
The structure of the convolution block.

The structure of the convolutional block is shown in Figure 3. Each convolutional block is composed of a convolutional layer, batch normalization, and activation layer. The dimensions of the signal will change after passing through the convolutional layer in order to extract suitable features. The vibration signal will pass through these layers in turn. Then, the flatten layer will re-adjust the dimension of the signal to one dimension again, and prepare for the next operation.

### 3.3. Wavelet Layer

#### 3.3.1. Wavelet Transform

For the wavelet transform of vibration signals, suppose the signal to be transformed is *x*(*t*), and the wavelet basis function Ψ(t) is defined as

(1) $$\Psi_{a\;,b}\left(t\right)=\;\frac{1}{\sqrt{\left|a\right|}}\Psi \left(\frac{t-b}{a}\right)$$

where a denotes the dilation factor and b denotes the translation factor. The expansion and contraction of the wavelet is characterized by the dilation factor a; and the displacement of the wavelet is characterized by the translation factor b.

The continuous wavelet transform is defined as:

(2) $$WT_{x}\left(a,b\right)=\frac{1}{\sqrt{\left|a\right|}}\int^{}x\left(t\right)\Psi \left(\frac{t-b}{a}\right)dt=〈x\left(t\right),\Psi_{a\;,b}\left(t\right)〉$$

From the definition of the inner product of the wavelet transform, it can be obtained that $WT_{x}\left(a,b\right)$ represents the projection of the signal x(t) on the wavelet basis function Ψ(t).

When performing those operations on a computer, the parameters a and b need to be discretized. Assuming $a=a_{0}^{m},b=nb_{0}a_{0}^{m}$,m,n∈Z, then

(3) $$\Psi_{n,m}\left(t\right)=\;\frac{1}{\sqrt{\left|a_{0}\right|}}\Psi \left(a_{0}^{-m}t-nb_{0}\right),\;m,n\in Z$$

In summary, the discrete wavelet transform is defined as:

(4) $$WT_{x}\left(a,b\right)=\frac{1}{\sqrt{\left|a\right|}}\int^{}x\left(t\right)\Psi \left(a_{0}^{-m}t-nb_{0}\right)dt$$

Wavelet transform has the characteristics of being flexible and changeable, and can carry out multi-scale analysis. By adjusting the dilation factor a and the translation factor b, the signal can be observed step by step from the whole to the part, and the signal can be analyzed in the time and frequency domain, which has been widely used in the field of signal analysis [46]. 

#### 3.3.2. Wavelet Layer Design

We designed a wavelet layer to further extract the features extracted from the previous convolutional block. Assuming that the input dimension of the wavelet layer is i and the output dimension is j, the output of the wavelet layer can be expressed as:

(5) $${\overset{\text{\textasciicircum{}}}{y}}_{i}=\Psi_{a,b}\left(\frac{\sum_{i-1}^{m}w_{\mathrm{ij}}x_{i}-b_{j}}{a_{j}}\right),j=1,2\ldots n,i=1,2\ldots m$$

where ${\hat{y}}_{i}$ denotes the output of the wavelet layer, Ψ is the activation function, $w_{ij}$ is the connection weight between the input layer and the output layer.$\;a_{j},\;b_{j}$ are the weights to be learned.

The wavelet layer inherits the advantages of the wavelet transform, which makes the neural network converge fast and avoids falling into the local optimum. It also has the characteristics of time-frequency local analysis [40].

In this paper, Morlet wavelet is used as the wavelet basis function Ψ:

(6) $$\Psi \left(x\right)=\cos\left(4x\right)\exp\left(-\frac{t^{2}}{2}\right)$$

### 3.4. Prototypical Layer

**Figure 4:**
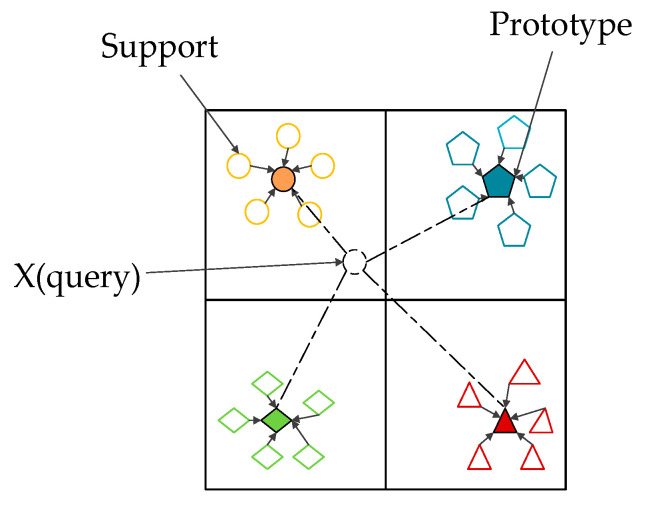
Prototypical layer in the 4-way 5-shot scenario.

Differing from the traditional neural network layer, the Prototypical layer can map its input to a feature space, and extract their “mean” to represent the prototype of the class. Using Euclidean distance as a distance metric, the training process of the model makes the distance of the same class of data to their prototypes the closest, while the distance to other prototypes becomes farther. During the test phase, the distance between the test data and the prototype data of each category is classified by softmax function to determine the labels of the test data. The classification principle of the prototypical layer is shown in Figure 4. A prototype $c_{k}$ must be calculated for each category. A mapping function $f_{\varphi }:{\mathbb{R}}^{D}\to {\mathbb{R}}^{M}$ is used to map the sample data of dimension *D* to the *M*-dimensional space. By mapping the *N* samples in the *k*-th class in support set, *N* points can be obtained in this *M*-dimensional space. Take the mean value of these *N* points as the prototype, namely $c_{k}$. In summary, $c_{k}$ can be calculated by the following equation:

(7) $$c_{k}=\frac{1}{\left|S_{k}\right|}\underset{\left(x_{i\;},\;y_{i\;}\right)\in S_{k}}{\sum }f_{\theta }\left(x_{i}\right)$$

where $f_{\theta }$ denotes a mapping function with learnable parameters θ, $S_{k}$ denotes the set of examples labeled with class *k*, $x_{i\;}\in {\mathbb{R}}^{D}$ is the *D*-dimensional feature vector of an examp and $y_{i\;}\in \left\{1,2,3\ldots k\right\}$. is the corresponding label [39].

### 3.5. Training of the Model

After the input data is mapped to the feature space by the prototypical layer, the distance between them is determined by the Euclidean distance:

(8) $$d\left(z,z^{\prime }\right)={\left|\left|z-z^{\prime }\right|\right|}^{2}$$

Assuming that the point after a query is mapped to the feature space is x, the prototypical layer will take the distance between x and each prototype, and use the softmax function to generate a probability distribution of x, namely:

(9) $$p_{\theta }\left(y=k|x\right)=\frac{\exp\left(-d\left(f_{\theta }\left(x\right),c_{k}\right)\right)}{\sum_{k^{,}}\exp\left(-d\left(f_{\theta }\left(x\right),c_{k^{\prime }}\right)\right)}$$

**Figure 5:**
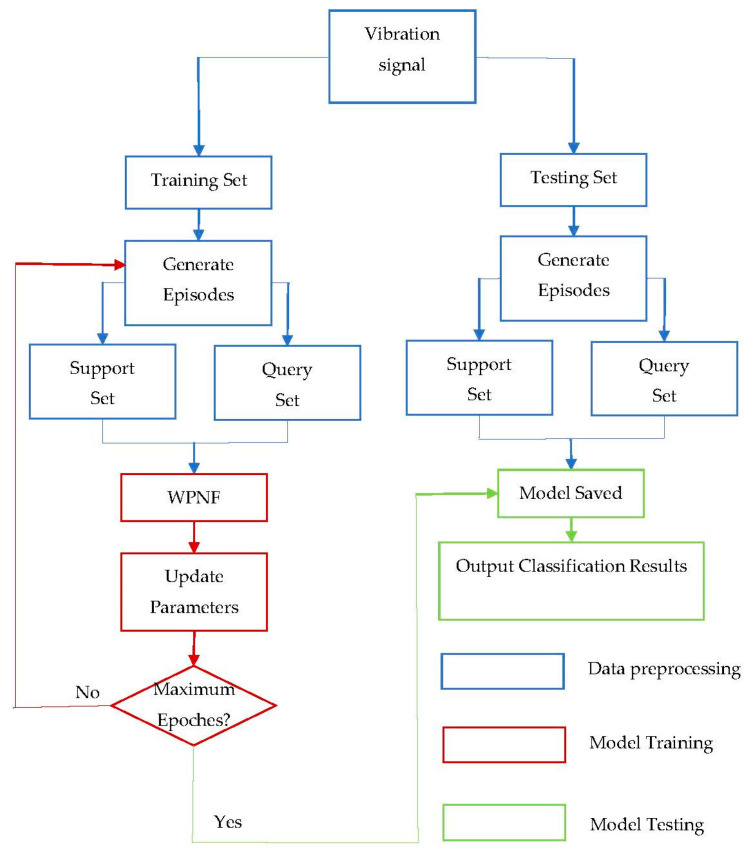
Flowchart of model training.

Flowchart of model training is shown in Figure 5. During the training process, each epoch is composed of several episodes. In each episode, the model randomly selects $N_{C}$ classes, and a very small number of samples in each class are selected as supports, and there are $N_{Q}$ samples left in each class as queries. The loss function is defined as the negative log-probability $J\left(\theta \right)=-logp_{\theta }\left(y=k|x\right)$ of the true label of the sample:

(10) $$J\left(x,\;c_{k}\right)=-log\frac{1}{N_{Q}}\overset{N_{Q}}{\underset{i=1}{\sum }}\frac{\exp\left(-d\left(f_{\theta }\left(x\right),c_{k}\right)\right)}{\sum_{k^{,}}\exp\left(-d\left(f_{\theta }\left(x\right),c_{k^{\prime }}\right)\right)}$$

The model uses momentum stochastic gradient descent (SGD) method to update the trainable parameters θ. The pseudocode of the algorithm for updating parameters through the SGD method is shown in Algorithm 1.
**Algorithm 1** Update the trainable parameter θ of WPNF via the stochastic gradient descent method of momentum**Require**: learning rate η, Momentum parameter α, Initial parameters θ, Initial speed v **For** epoch **to** set value, **do**:
  **For** episode **to** set value, **do**:
   Randomly take m samples $\left\{x^{\left(1\right)},x^{\left(2\right)},x^{\left(3\right)}\ldots x^{\left(m\right)}\right\}$ from the query set, and their true labels are $c_{k}{}^{\left(i\right)}$
   Compute gradient of the samples: $g\leftarrow \frac{1}{m}\nabla_{\theta }\underset{i}{\sum }J\left(x,\;c_{k}\right)$   Compute speed update: v←αa−ηg   Update parameters: θ← θ+v
  **End For** **End For**

## 4. Experiments 

### 4.1. Description of Experimental Data

**Figure 6:**
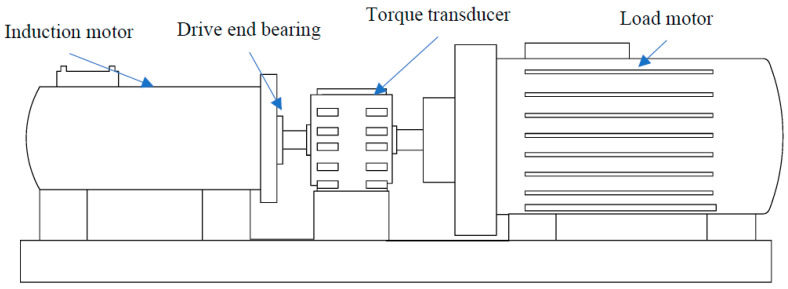
Case Western Reserve University Bearing Test Bench.

**Figure 7:**
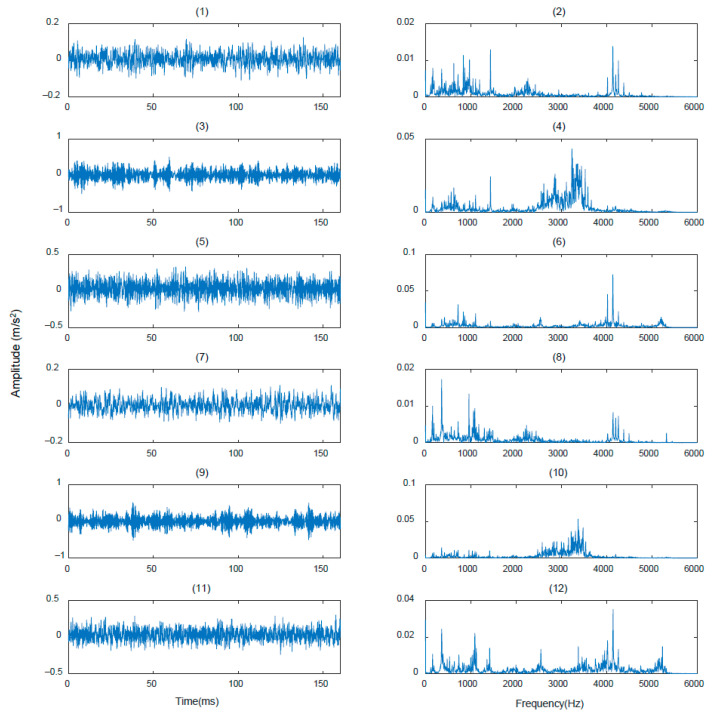
Visualization of a part of training data.

We use the bearing data set published by Case Western Reserve University (CWRU) for experiments [47]. The Case Western Reserve University Bearing Test Bench is shown in Figure 6. As a way of meta-learning, WPNF training needs to use a large number of classes to learn the ability of classification. Therefore, the training process in this paper imitated the work of Snell et al. [30]. In order to expand the number of classes, the heath states were subdivided, we used data with a sampling frequency of 12k HZ, and the bearing data was divided into different measuring points (drive end, fan section, base), faulty bearing position (drive end, fan section), fault diameter (0.007 inch, 0.014 inch, 0.021 inch, 0.028 inch), motor load (0HP, 1HP, 2HP, 3HP) and failure mode (inner race fault, ball fault, outer race fault). We subdivided them into a total of 200 classes, of which 170 classes were used for training and 30 classes were used for testing. There are only 20 samples for each class to be divided into support and query. We also selected part of the data for visualization, as shown in Figure 7. Among them (1), (2), (3) are the data measured from the base, drive end, and fan end when the ball of the drive end bearing is faulty and the damage diameter is 0.07 inch when the motor load is 0 HP. Next, (4), (5), (6) are their spectrograms after FFT transformation. It can be seen that even with the same fault form, with different measuring points, the frequency domain characteristics will be very different. Regarding (7), (8), (9), these are the data measured from the base, drive end and fan end when the drive end bearing rolling failure occurs when the motor load is 1HP, and the damage diameter is also 0.07 inch. Finally, (10), (11), (12) are their spectrograms after FFT transformation. It can be seen that the motor load will also affect the characteristics of the signal in the frequency domain. In summary, it can be found that the frequency domain signal is more sensitive to the health of the bearing than the time domain signal, so it is very suitable to be used as a supplement to the original signal and sent to the neural network for training to improve the accuracy and reliability of the neural network.

### 4.2. Hyperparameters Setting of WPNF

There are 2048 points in each sample. In order to improve the anti-noise performance of the model, we used wide convolution kernels in the first convolution block [15]. Therefore, as is shown in convolution blocks 1 and 5 both use 64 × 1 kernels, and the rest of the convolution blocks use ordinary convolution kernels (3 × 1). The number of convolution kernels in the convolution block is 64. We used maximum pooling as the pooling method and the kernel size was set to 2. The epoch was set to 200, and the episodes contained in each epoch were 100. A test was performed after each epoch, also composed of 100 episodes, we then take their average accuracy rate as the accuracy rate of this epoch. We used the 10-ways scenario during training stage and the 5-ways scenario during testing stage, and saved the model with the highest test accuracy as the best model (Table 1).

### 4.3. Several Models for Comparison

(1). SVM: Support Vector Machine (SVM) is a pattern recognition method based on statistical learning theory. Although SVM is essentially a two-class classifier, it can be extended to a multi-class classifier. Common methods include one-versus-rest and one-versus-one. This experiment extracted five features of data peak value, peak-to-peak value, root mean square, kurtosis, and margin, and sent the samples to the vector machine for classification. 

(2). Deep Convolutional Neural Networks with Wide First-layer Kernels (WDCNN) [15]: A traditional machine learning model that can directly classify data without any preprocessing. A large number of samples are needed as a training set.

(3). Pro Net: The prototypical network mentioned in [39]. It only changes the input channel of the prototype network to one dimension.

(4). WPNF: The wavelet-prototypical network based on fusion of time and frequency domain which can fuse the time domain and frequency domain information of the original signal, and introduce a wavelet layer to improve the generalization and reliability of the model.

### 4.4. Comparison of Experimental Results

**Figure 8:**
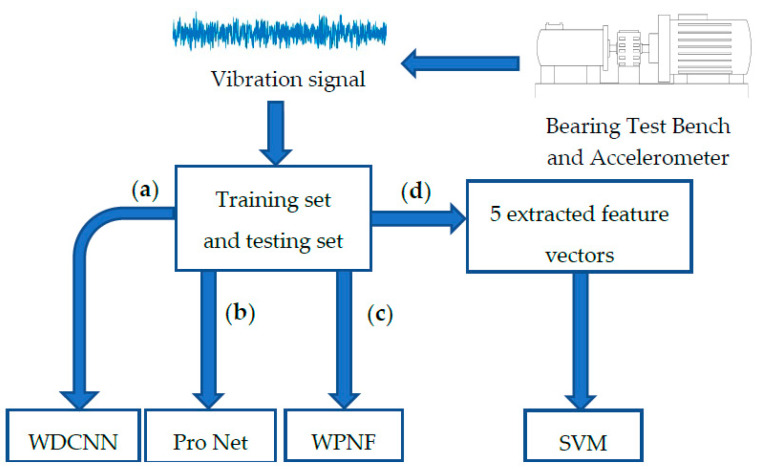
Flow chart of the experiment: (**a**) Deep Convolutional Neural Networks with Wide First-layer Kernels (WDCNN); (**b**) Pro Net; (**c**) The proposed method (the wavelet-prototypical network based on fusion of time and frequency domain WPNF); (**d**) Support Vector Machine (SVM).

**Figure 9:**
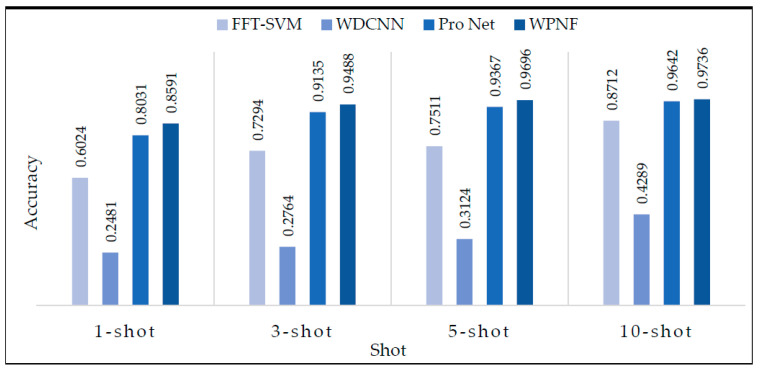
Comparison results of accuracy of models.

We performed comparative experiments between the above models under the same data set and used the same equipment in the scenarios of 5-way 1-shot, 5-way 3-shot, 5-way 5-shot, and 5-way 10-shot. In order to ensure the accuracy of the experimental results, we repeated the experiment under each scenario 10 times, and took the average accuracy rate as the final accuracy rate. The block diagram of our experiment is shown in the Figure 8. The final result is shown in Table 2:

It can be seen from Figure 9 that the accuracy of the four models will increase as the number of training samples increases. However, as traditional machine learning methods, whether it is SVM or WDCNN, the accuracy rate is disappointing. This is because traditional machine learning methods can only achieve good results when training samples are sufficient. With the increase of training samples, the accuracy rate of WDCNN becomes the lowest. This is because the network structure of WDCNN is too complex and the number of training samples is insufficient, which leads to serious overfitting. As a meta-learning method, the accuracy rate of Pro Net and WPNF has always been maintained at a high level, even in the 1-shot scenario. The classification accuracy rate of WPNF surpassed Pro Net in these four scenarios. The improvement is most obvious in the 1-shot scenario, which is 5.6%. With the increase of training samples, the gap of the two models gradually decreased, respectively 3.53%, 3.29%, 0.94%. This shows that in the few-shot scenario, WPNT is superior to traditional machine learning models or meta-learning methods.

#### 4.4.1. Visualization of the Performance of WPNF and Pro Net

**Figure 10:**
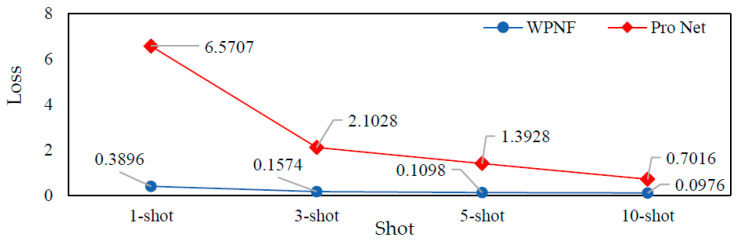
Comparison results of loss of the wavelet-prototypical network based on fusion of time and frequency domain (WPNF) and Pro Net.

**Figure 11:**
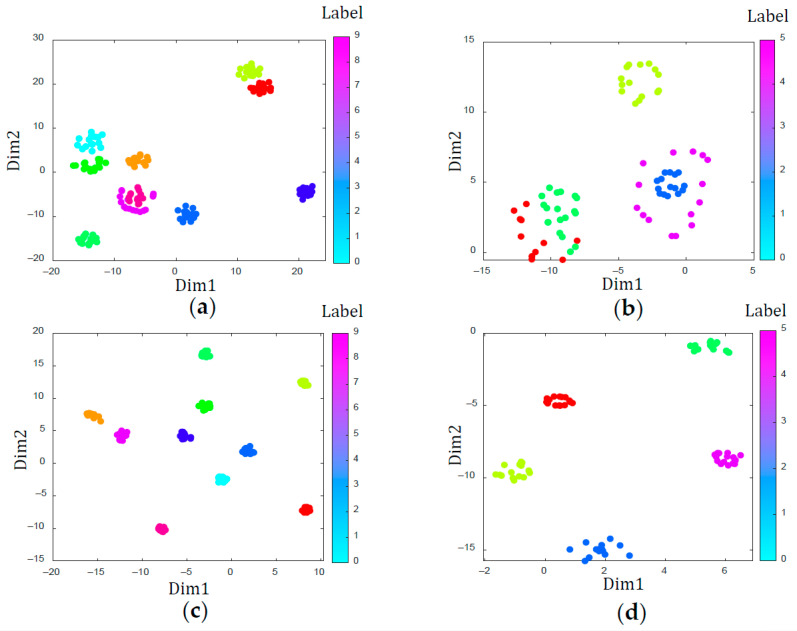
Visualization of the features extracted by Pro net and the wavelet-prototypical network based on fusion of time and frequency domain (WPNF) via t-SNE. (**a**) Pro net on the training set; (**b**) Pro net on the test set; (**c**) WPNF on the training set; (**d**) WPNF on the test set.

In order to compare WPNF and Pro Net more intuitively, we visualized the training results of these two models in some scenarios. Figure 10 is a comparison diagram of the loss value of the two models with different number of samples. Figure 11 is the result of taking out the features extracted from the last hidden layer of the two models during the training phase and the testing phase, and visualizing them using the t-SNE method. 

WPNF and Pro Net use the same loss function, so the losses of the two can be compared visually. As can be seen from Figure 10, the loss of WPNF in the test is an order of magnitude lower than that of Pro Net. In the 10-shot scenario, even if the accuracy difference between the two is less than 1%, the difference in their loss is very large, the loss of WPNF is only 14% of Pro Net’s. This shows that no matter which scenario, the performance of WPNF is more stable and reliable.

We use t-SNE (t-distributed stochastic neighbor embedding) [48] to visualize the performance of WPNF and Pro Net on the training set and test set respectively, where the training scenario was 10-way 5-shot and the test scenario was 5-way 5-shot. The result is shown in Figure 11. It can be seen that both WPNF and Pro Net achieved very good results on the training set, but the results of Pro Net on the test set were not satisfactory. Although in this scenario, the accuracy of Pro net on the test set is only 3.29% lower than WPNF, its reliability and generalization ability are far inferior to WPNF.

#### 4.4.2. Comparison of WPNF and Several Other Models

We made the confusion matrix of the several models mentioned in this paper to better compare the stability and reliability of these models. In order to increase the credibility of the results, we repeated the experiments for each model. For Pro net and WPNF, we randomly selected four episodes during the test phase and made the corresponding confusion matrix. For SVM and WDCNN, the experiment was repeated four times, and the confusion matrix of the four test results was made. 

**Figure 12:**
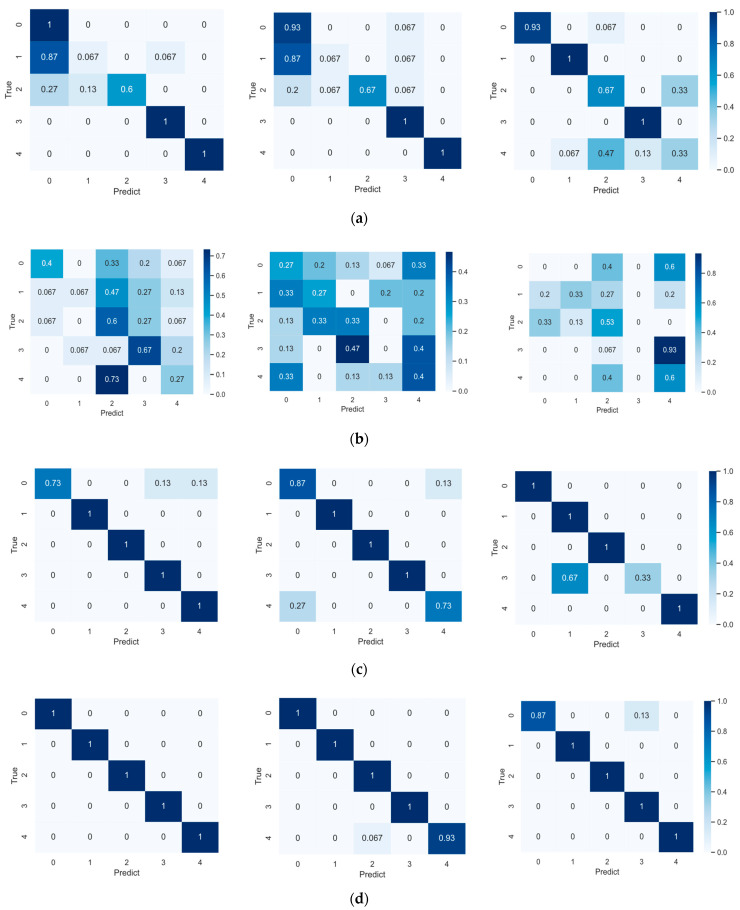
Confusion matrix of several models mentioned in the paper (each model is repeated 3 times) (**a**) Support Vector Machine (SVM); (**b**) Deep Convolutional Neural Networks with Wide First-layer Kernels (WDCNN); (**c**) Pro net; (**d**) the wavelet-prototypical network based on fusion of time and frequency domain (WPNF).

The confusion matrix of the above four models in the 5-way 5-shot scenario is shown in Figure 12. It is not difficult to see that although SVM has certain learning ability, the upper limit of classification effect is low, and WDCNN almost loses its classification ability in the few-shot scenario. This is because the model structure of WDCNN is very complicated, and there are too many parameters to be trained. In the case of insufficient sample size, it cannot give full play to its advantages, and serious overfitting occurs. The results of WDCNN are almost random. In contrast, Pro net and WPNF both have good stability. This shows that the model based on meta-learning can indeed learn the ability of learning, not just limited to a certain classification task, and the performance of Pro net is more stable than WPNF and less error-prone.

## 5. Discussion

The failure of rotating equipment can bring huge losses to a business. Therefore, the method of condition monitoring for rotating machinery is particularly important. At present, there are mainly three methods for intelligent fault detection: acoustic analysis, vibration analysis, and thermal images analysis. Among them, acoustic analysis and vibration analysis are relatively similar, because both sound signals and vibration signals are one-dimensional signals, and during manual analysis, both of them need to obtain the frequency spectrum of the signal through FFT and find the characteristic frequency of the corresponding fault for further analysis. Both can also be used to directly train an end-to-end one-dimensional neural network without any preprocessing. In contrast, vibration signals have less noise, so the accuracy rate is higher than acoustic signal analysis. But the acquisition of acoustic signals is less difficult, because the acoustic sensor does not need to be glued to the machine, so portability is better than that of an acceleration sensor. Thermal images analysis requires a thermal camera to obtain two-dimensional thermal pictures. For manual analysis, thermal images are more intuitive than acoustic signals and vibration signals, and it is easier to locate machine faults. The data volume of thermal images is relatively large, usually 640×480 pixels, and one sample of a one-dimensional acoustic signal or vibration signal usually has 1024 or 2048 points. Therefore, when the structure of the neural network is more complex and the number of samples used to train the neural network is sufficient, thermal images analysis can achieve a high accuracy rate, but it is difficult to collect enough samples for each type of fault in practical applications.

**Figure 13:**
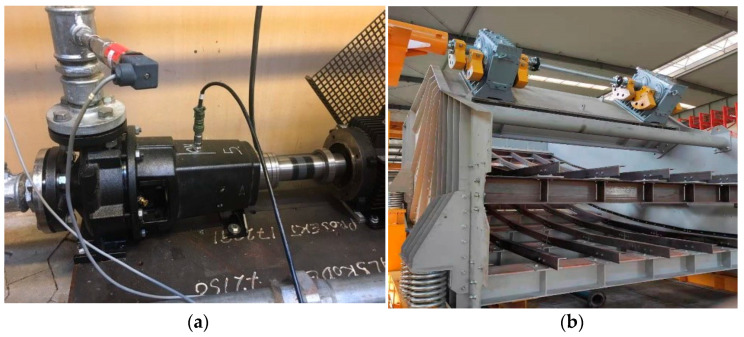
Machines that can apply the wavelet-prototypical network based on fusion of time and frequency domain (WPNF) for fault diagnosis: (**a**) Centrifugal pump, (**b**) Vibrating screen.

After comparing the advantages and disadvantages of these three methods, we chose to use vibration signals to verify our model, compared with several mainstream models, and the proposed model achieved good results. WPNF can be applied to any rotating equipment that can collect vibration signals, not just the fault diagnosis of bearings. Our future work will verify the application of WPNF on different rotating machinery (such as centrifugal pumps, vibrating screens, etc., as shown in Figure 13), and optimize WPNF according to the characteristics of the application scenarios.

## 6. Conclusions

We proposed a wavelet-prototypical network based on fusion of time and frequency domain (WPNF) that can be used to identify the health status of rotating equipment in few-shot scenarios in this paper. It can accurately identify new categories that have never been seen in the training process, and each of the new category requires very few samples. In order to test the proposed model, we conducted experiments on the model using the bearing data set publicly available by Case Western Reserve University, and compared it with other machine learning models, such as SVM, Pro Net, and WDCNN. The experimental results show that whether in the scenarios of 1 shot, 3 shot, 5 shot, or 10 shot, the proposed model achieved the best results. As a traditional machine learning model, the classification accuracy of SVM and WDCNN in few shot scenarios was not satisfactory, with 60.24%; 72.94%; 75.11%; 87.12% and 24.81%; 27.64%; 31.24%; 42.89%. As meta-learning methods, Pro Net and WPNF both achieved good accuracy. However, in the four scenarios, the classification accuracy of WPNF was 5.6%, 3.53%, 3.29%, and 0.94% higher than Pro Net respectively. This shows that the proposed WPNT is better than Pro Net and proves the feasibility of the proposed model.

The proposed WPNT can theoretically be applied to fault diagnosis of all mechanical equipment that can collect vibration signals, not just bearings. In future research, we will study the application of WPNT in the fault detection of different mechanical equipment, and find a method that can automatically optimize WPNF according to the characteristics of the vibration signal of different rotating machinery.

## Figures and Tables

**Table 1:** Hyperparameters setting of the wavelet-prototypical network based on fusion of time and frequency domain (WPNF).

Name	Filters	Kernel Size/Stride (Convolution)/Stride (Pooling)	Input Size	Output Size	Activation Function
convolutional block1	64	64 × 1/1 × 1/2 × 1	1 × 2048	64 × 992	Relu
convolutional block2	64	3 × 1/1 × 1/2 × 1	64 × 992	64 × 495	Relu
convolutional block3	64	3 × 1/1 × 1/2 × 1	64 × 495	64 × 246	Relu
convolutional block4	64	3 × 1/1 × 1/2 × 1	64 × 246	64 × 122	Relu
convolutional block5	64	64 × 1/1 × 1/2 × 1	1 × 1024	64 × 480	Relu
convolutional block6	64	3 × 1/1 × 1/2 × 1	64 × 480	64 × 239	Relu
convolutional block7	64	3 × 1/1 × 1/2 × 1	64 × 239	64 × 118	Relu
convolutional block8	64	3 × 1/1 × 1/2 × 1	64 × 118	64 × 58	Relu
Wavelet layer	/	/	11520	5120	Morlet
Prototypical layer	/	/	5120	5120	Softmax

**Table 2:** Comparison of accuracy of models.

	SVM	WDCNN	Pro Net	WPNF
**1-shot**	0.6024	0.2481	0.8031	0.8591
**3-shot**	0.7294	0.2764	0.9135	0.9488
**5-shot**	0.7511	0.3124	0.9367	0.9696
**10-shot**	0.8712	0.4289	0.9642	0.9736

## Data Availability

The data used to support the findings of this study are available at: https://csegroups.case.edu/bearingdatacenter/pages/download-data-file (accessed on 17 February 2021).
